# Elevated Pancreatic Enzymes in ICU Patients With COVID-19 in Wuhan, China: A Retrospective Study

**DOI:** 10.3389/fmed.2021.663646

**Published:** 2021-08-17

**Authors:** Peili Ding, Bin Song, Xuelin Liu, Xing Fang, Hongliu Cai, Dingyu Zhang, Xia Zheng

**Affiliations:** ^1^Department of Critical Care Medicine, The First Affiliated Hospital, Zhejiang University School of Medicine, Hangzhou, China; ^2^Department of Tuberculosis and Respiratory Disease, Jinyintan Hospital, Wuhan, China; ^3^Research Center for Translational Medicine, Wuhan Jinyintan Hospital, Wuhan, China; ^4^Joint Laboratory of Infectious Diseases and Health, Wuhan Institute of Virology and Wuhan Jinyintan Hospital, Chinese Academy of Sciences, Wuhan, China

**Keywords:** pancreatic enzymes, amylase, lipase, pancreatitis, COVID-19

## Abstract

**Background:** Pancreatic enzyme elevation has been reported in patients with COVID-19 during the pandemic. However, with the shortage of medical resources and information, several challenges are faced in the examination and treatment of this condition in COVID-19 patients. There is little information on whether such condition is caused by pancreatic injury, and if this is a warning sign of life threatening complications like multiple organ failure in patients. The objective of this study is to explore the relationship between elevated pancreatic enzymes and the underlying risk factors during the management of COVID-19 patients.

**Method:** A total of 55 COVID-19 patients admitted to the intensive care unit (ICU) of Wuhan Jinyintan hospital from January 1 to March 30, 2020 were enrolled in this study. All participants underwent transabdominal ultrasound imaging to assess their pancreas.

**Results:** Out of the 55 patients, three patients had pancreatitis, 29 (52.7%) with elevated pancreatic enzymes, and 23 (41.8%) without. The most common symptoms of patients with COVID-19 were fever and cough. There was no statistical difference in most baseline characteristics except myalgia on admission. Compared with those having normal enzyme levels, patients with elevated pancreatic enzymes had higher rates of mortality (79.3 vs. 52.2%; *P* = 0.038), and lower rates of discharge (20.7 vs. 47.8%; *P* = 0.038). Patients with elevated enzymes had higher incidence of mechanical ventilation (*P* = 0.004) and kidney injury (*P* = 0.042) than patients without elevated pancreatic enzymes. The results of multivariable logistic analysis showed that the odds ratio were 10.202 (*P* = 0.002) for mechanical ventilation and 7.673 (*P* = 0.014) for kidney injury with the elevated enzymes vs. the normal conditions.

**Conclusions:** The findings show that the incidences of pancreatic enzymes elevation are not low in critical COVID-19 patients and only a few of them progressed to acute pancreatitis (AP). Increased pancreatic enzymes levels is associated with poor prognosis in COVID-19 patients. In addition, the kidney injury and oxygenation degradation are associated with the pancreatic enzymes elevation in COVID-19 patients.

## Introduction

There was reported outbreak of a typical pneumonia-like respiratory disease in Wuhan, Hubei, China, that quickly spread all over the country and the world. The outbreak was described as a pandemic on March 11, 2020 by the World Health Organization. Through deep sequencing of respiratory specimens, it was later confirmed as an acute respiratory infectious disease caused by a novel coronavirus 2019 (SARS-coronavirus 2) (1). SARS-coronavirus 2 (SARS-CoV-2) belong to the β coronavirus genes, similar to the severe acute respiratory syndrome coronavirus (SARS-COV) and Middle East respiratory syndrome coronavirus (MERS-COV). Similar to SARS-COV and MERS-COV, SARS-CoV-2 also enters the human body cells through spike protein to combine with the angiotensin-converting enzyme-2 (ACE-2) receptor (2–4).

Both SARS-CoV-2 and SRAS-COV have spike proteins sharing a high degree of homology in sequences and a number of amino acids (5, 6). However, their genetic characteristic is different in some aspects and their nucleic acid homology is <80% (2). The SARS-CoV-2 has a higher rate of spreading from one person to another than SRAS-COV. According to previous studies, it is suspected that SARS-CoV-2 has higher and more efficient ability to identify human ACE2 receptor than SARS-COV. It binds with ACE2 receptors more strongly and this facilitates its quick entry to human cells (7). Human alveolar epithelial type-II cells express abundant ACE-2 receptors to facilitate the virus enter the lung. This makes the lung to be the most vulnerable target organ to the virus (8, 9).

The ACE2 is not only abundantly expressed in lung and small intestine tissues but also in endothelial cells and smooth muscle cells of almost all human organs (10). In 2003, the infectious pneumonia SARS-COV virus was found in several human organs including lung, kidney, intestine, and pancreas (10). Irina et al. demonstrated the prominent expression of ACE-2 in the pancreatic ductal and microvascular epithelium. This makes the tissues to be a more potential targets of the coronavirus and subsequent pancreatic injury (11). Amer-Hadi et al. reported the presentation of acute pancreatitis as a complication caused by SARS-CoV-2 in two of the three members of the same family with the coronavirus disease 2019 (COVID-19) (12).

Two studies (13, 14) have reported different cases of the COVID-19 which developed into severe acute pancreatitis (AP). Interestingly, several patients had extremely high lipase levels but not diagnosed with pancreatitis. This was confirmed by abdominal imaging in our ICU clinical work during the COVID-19 epidemic in Wuhan, China. The interesting phenomenon in these studies have been puzzling: Did it also occurred on other COVID-19 patients? Was the incidence high or just casual? Was this a warning sign of multiple organ failure? Were there several risk factors for the pancreatic enzymes elevation?

There are several research studies on the complication of elevated pancreatic enzymes in ICU COVID-19 patients. Wang et al., *for example*, reviewed lipase levels and described the incidence of pancreas injuries in 52 patients with COVID-19 (15). However, they did not perform abdominal imaging on the patients that would be important for pancreas assessment. They also did not analyze the possible risk factors of the pancreatic enzymes elevation. To understand the relationship between the SARS-CoV-2 and the clinical phenomenon of elevated pancreatic enzymes, this study reviewed relevant clinical data to explore the phenomenon and the possible risk factors behind it.

## Method

### Data Collection

**Figure 1:**
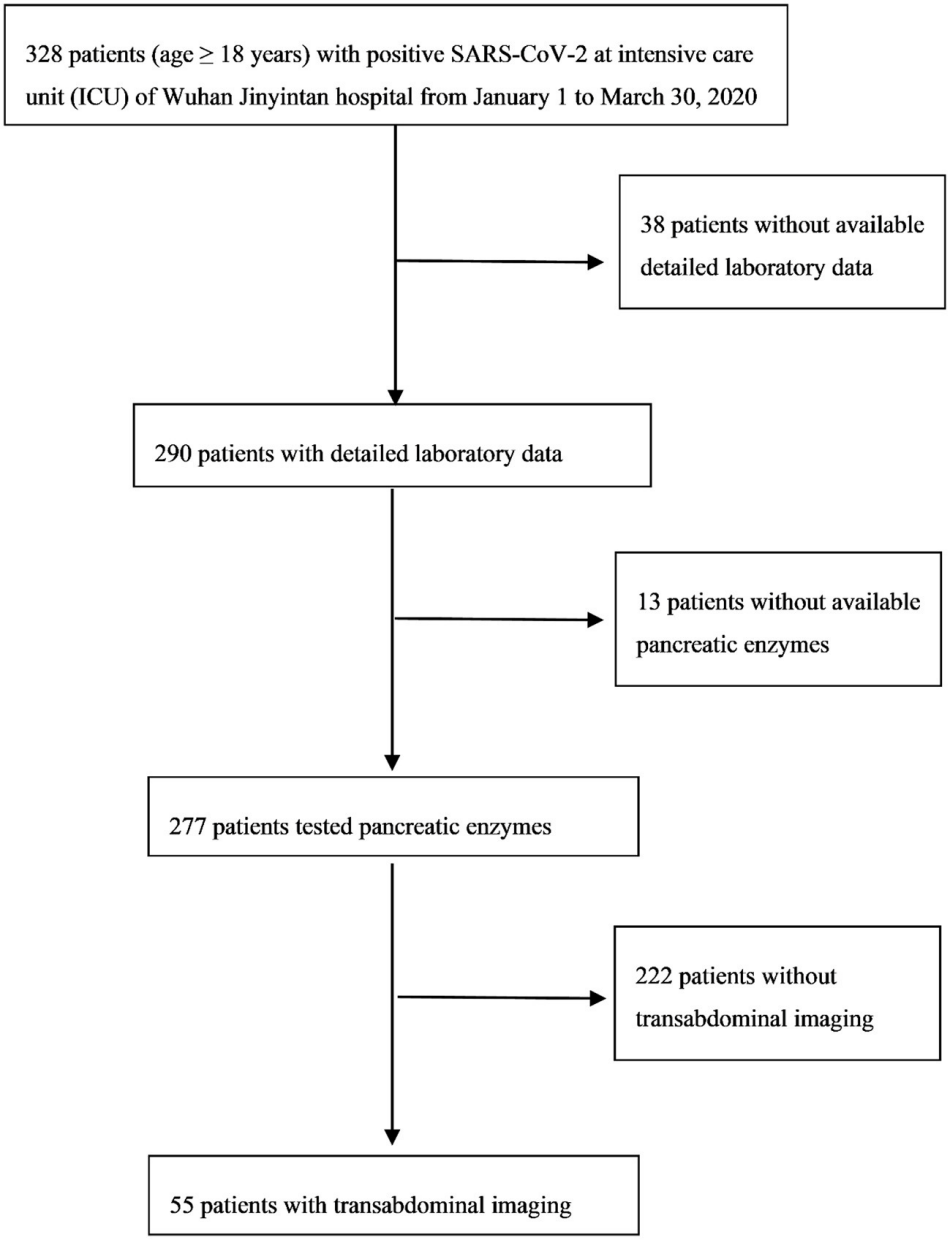
Recruitment flowchart.

In our retrospective research, the inclusion criteria were all critical patients (age ≥18 years) with positive SARS-CoV-2 and in the intensive care unit (ICU) of Wuhan Jinyintan hospital from January 1 to March 30, 2020 (*n* = 328). Detailed laboratory data of 290 patients was available. Pancreatic lipase (normal range between 8 and 78 U/L) or amylase (normal range between 35 and 135 U/L) were tested in 277 patients. Transabdominal ultrasound imaging was conducted on 55 patients (Figure 1). This research study was approved by the Medical ethics committee of the Wuhan Jinyintan hospital and the patients were followed up to discharge or death.

### Research Object

The respiratory tract or blood samples from patients were tested positive for the new coronavirus nucleic acid by real-time reverse transcription-polymerase chain reaction (RT-PCR), or specimen viral gene sequencing was highly homologous to the known new coronavirus (16). The seventh edition (17) of the COVID-19 diagnosis and treatment plan was used to classify the clinical severity of the new coronavirus pneumonia. The specific classification criteria were as follows: ordinary type (with fever, respiratory symptoms, and pneumonia manifestations on imaging); severe type [met any of the following: (1) breathing rate ≥30/min; (2) oxygen saturation ≤ 93% at rest; (3) oxygenation index ≤ 300 mmHg; (4) lung imaging shows that the lesion has progressed significantly more than 50%]; critical type [met any of the following: (1) respiratory of failure requires mechanical ventilation; (2) shock; (3) other organ failure requires ICU monitoring and treatment]. According to the revised Atlanta Classification, pancreatitis was defined as at least 2 of the following 3 items: (1) abdominal pain; (2) serum lipase and/or amylase at least 3 times more than the upper limit of normal (>3 × ULN); (3) imaging characteristic findings of acute pancreatitis on contrast-enhanced computed tomography (CT), transabdominal ultrasonography, or magnetic resonance imaging (MRI) (18). Kidney injury was defined according to the Kidney Disease: Improving Global Outcomes (KDIGO) (19). Shock was defined as life-threatening acute circulatory failure accompanied by inadequate cellular oxygen utilization (20, 21). It was noted that the oxygenation deterioration and the judgment of whether to change the mode of oxygen therapy or the need for invasive endotracheal intubation were at the discretion of the ICU clinicians.

### Statistical Method

The continuous data of this study were described as mean ± S.E.M (standard error of mean), whereas the categorical data were described as percentages. Comparisons of the categorical data were appropriately conducted using Chi-square tests or Fisher's exact tests. Single-factor analysis of variance or Kruskal-Wallis H tests were performed for the appropriate comparisons of the continuous data. Univariate logistic analysis and multivariate logistic analysis were used to quantify the associations between pancreatic enzymes elevation with relevant risk factors. The significant difference was reported at *P* < 0.05.

## Results

### Baseline Clinical Characters

A total of 55 patients were enrolled in the study. Out of the 55 critically ill COVID-19 patients, there were three patients with pancreatitis, 29 with elevated pancreatic enzymes, and 23 within the normal range of pancreatic enzymes. The enrolled patients were aged between 29 and 79 years. Eighteen (32.7%) were women, the median age was 63 years and the average age was 61 years. The age of the three patients with pancreatitis was 55, 45, and 29 years (mean, 43 years). The average age of patients with and without elevated pancreatic enzymes was 63, 61 years, respectively (Table 1). It was observed that the most common clinical symptoms were fever and cough (Table 2). It was reported that among the patients with pancreatitis, one patient had hyperlipidemia before the study. None of the patients in all the three groups had stomachache. Comparative analysis show that the patients with elevated pancreatic enzymes had a higher incidence of diarrhea, myalgia, vomit, and previous history including hypertension and diabetes on admission than those without elevated enzymes. The patients in the three groups show significant differences in myalgia after admission (*P* = 0.02).

**Table 1:** Clinical characteristics of patients with COVID-19.

**Variable**	**Patients of COVID-19 with pancreatitis**				**Patients of COVID-19 with elevated** ** pancreatic enzymes** ** (*N* = 29)**	**Patients of COVID-19 without** ** elevated enzymes** ** (*N* = 23)**	***P***
	**P1**	**P2**	**P3**	**Overall**			
Age (mean)	55	45	29	43 ± 13	63 ± 12	61 ± 11	0.18
Sex, male n (%)	N	Y	N	2(66.7)	18(62.1)	17(73.9)	0.72
Epidemiology	N	N	N	0(0)	0(0)	0(0)	–
**Comorbidities**, ***n*****(%)**							
Hypertension	N	N	N	0(0)	13(44.8)	9(39.1)	0.41
Diabetes	N	N	N	0(0)	6(20.7)	4(17.4)	1
COPD	N	N	N	0(0)	0(0)	0(0)	–
Cardiac disease	Y	N	N	1(33.3)	2(6.9)	2(8.7)	0.37
Chronic Renal disease	N	N	N	0(0)	1(3.4)	2(8.7)	0.64
Carcinoma	N	N	N	0(0)	1(3.4)	3(13)	0.46
HCV	N	N	N	0(0)	0(0)	0(0)	–
HIV	N	N	N	0(0)	0(0)	0(0)	–
Hyperlipidemia	Y	N	N	1(33.3)	0(0)	0(0)	0.05

*COPD, chronic obstructive pulmonary disease; HIV, human immunodeficiency virus; HCV, hepatitis C virus. The enrolled patients were aged between 29 and 79 years. Eighteen (32.7%) were women, the median age was 63 years and the average age was 61 years*.

**Table 2:** Chief complaints of patients with COVID-19.

**Variable**	**Patients of COVID-19 with pancreatitis**				**Patients of COVID-19 with elevatedpancreatic enzymes(*N* = 29)**	**Patients of COVID-19 without elevated enzymes (*N* = 23)**	***P***
	**P1**	**P2**	**P3**	**Overall**			
**Chief complaints on admission**, ***n*****(%)**							
Fever	Y	Y	Y	3(100)	27(93.1)	20(87)	0.73
Cough	N	Y	Y	2(66.7)	21(72.4)	19(82.6)	0.50
Expectoration	N	Y	Y	2(66.7)	10(34.5)	12(52.2)	0.33
Fatigue	N	N	N	0(0)	15(51.7)	9(39.1)	0.24
Nausea	N	N	N	0(0)	2(6.9)	2(8.7)	1
Vomit	N	N	N	0(0)	1(3.4)	0(0)	1
Diarrhea	N	N	N	0(0)	2(6.9)	0(0)	0.55
Stomachache	N	N	N	0(0)	0(0)	0(0)	–
Myalgia	Y	N	Y	2(66.7)	3(10.3)	1(4.3)	0.02
Headache	N	Y	N	1(33.3)	1(3.4)	0(0)	0.11
**Previous history**							
Smoking history	N	N	N	0(0)	2(6.9)	7(30.4)	0.09
Drinking history	N	N	N	0(0)	1(3.4)	4(17.4)	0.28

### Baseline Laboratory Results

We collected laboratory test results of 55 COVID-19 patients on admission (Table 3). According to the results, the patients with pancreatitis had increased leukocytes, neutrophils, NLR, and PLR. Further, there were no significant differences in the inflammatory indicators, such as CRP, PCT, ESR, and IL-6, blood coagulation functions, as well as blood biochemistry among the 55 patients with COVID-19.

**Table 3:** Laboratory results of patients with COVID-19.

**Variable**	**Patients of COVID-19 with pancreatitis**				**Patients of COVID-19 withelevated Pancreatic enzymes(*N* = 29)**	**Patients of COVID-19 without elevated enzymes (*N* = 23)**	***P***
	**P1**	**P2**	**P3**	**Overall**			
**Blood cytology(10** ^**9**^ **/L)Mean** **±** **SD**							
Leukocytes (3.5–9.5)	33.48	12.64	7.84	18.0 ± 13.6	11.0 ± 5.5	8.40 ± 3.6	0.09
Neutrophils (1.8–6.3)	32.55	11.89	7.23	17.2 ± 13.5	12.7 ± 15.3	7.30 ± 3.5	0.17
Lymphocyte (1.1–3.2)	0.4	0.44	0.47	0.44 ± 0.04	0.72 ± 0.6	0.70 ± 0.2	0.55
Platelets (125–350)	234	220	114	189.3 ± 65.6	201 ± 116.9	190.20 ± 77.3	0.92
NLR	81.4	27.0	15.4	41.3 ± 35.2	28.7 ± 40.5	12.40 ± 7.5	0.06
PLR	585	500	242.3	442.5 ± 178.3	437.8 ± 493.2	320.00 ± 182.0	0.53
**Inflammatory indicatorsMean** **±** **SD**							
CRP (0–5 mg/L)	122.5	160	43.8	108.8 ± 59.3	103.4 ± 53.5	95.3 ± 57.2	0.13
PCT (<0.5 ng/ml)	0.24	0.2	0.05	0.16 ± 0.1	0.81 ± 2.5	0.32 ± 0.55	0.66
ESR (0–20 mm/h)	37	115	65	72.3 ± 39.5	54.2 ± 22.0	62.3 ± 26.4	0.22
IL-6 (0–7)	13.7	12.8	9.2	11.9 ± 2.4	12.9 ± 8.3	10.10 ± 4.3	0.88
**Blood biochemistryMean** **±** **SD**							
ALB (40–55 G/L)	30.8	26.7	27	28.2 ± 2.3	30.9 ± 9.1	28.90 ± 4.8	0.59
ALT (7–40 U/L)	129	48	25	67.3 ± 54.6	66.00 ± 107.9	38.30 ± 40.2	0.49
AST (13–35 U/L)	65	35	32	44 ± 18.2	60.9 ± 50.3	41.70 ± 18.1	0.21
γ-GT (7–45 U/L)	159	112	50	107 ± 54.7	92.40 ± 84.4	89.70 ± 138.6	0.97
ALP (50–135 U/L)	108	122	44	91.3 ± 41.6	112.70 ± 56.0	104.3 ± 58.5	0.76
TB (0–21 umol/L)	12	24.6	9.8	15.5 ± 8.0	22.10 ± 18.7	13.20 ± 7.3	0.162
Cr (41–81 umol/L)	31.8	39	81.7	50.8 ± 27.0	145.40 ± 262.7	184.50 ± 276.5	0.68
LDH (120–250 U/L)	672	209	337	406 ± 239.1	697.10 ± 635.46	483.00 ± 213.6	0.24
CK (40–200 U/L)	123	258	35	138.7 ± 112.3	234.0690 ± 384.8	208 ± 185.1	0.86
CK-MB (0–24 U/L)	17	12	7	12 ± 5	19.60 ± 22.1	19.80 ± 15.0	0.79
TnT (0–28 Pg/ml)	127.8	7.7	5.2	46.9 ± 70.1	1418.9 ± 5599.1	76.00 ± 179.9	0.48
**Coagulation functionsMean** **±** **SD**							
PT (10.5–13.5S)	10.1	13.4	9.5	11 ± 21	13.70 ± 5.3	17.70 ± 19.3	0.47
APTT (21–37S)	19.1	27.3	17.5	21.3 ± 5.3	30.30 ± 7.8	34.30 ± 18.4	0.23
INR (0.8–1.2)	0.87	1.18	0.82	0.96 ± 0.2	1.20 ± 0.6	1.43 ± 1.6	0.63
Fibrinogen (2–4 g/l)	4	7.2	1.1	4.1 ± 3.1	4.30 ± 1.8	5.10 ± 2.1	0.3

*NLR, rate of neutrophils and lymphatic; PLR, rate of platelets and lymphatic; CRP, C-reactive protein; PCT, procalcitonin; ESR, erythrocyte sedimentation rate; IL-6, interleukin 6; ALB, albumin; ALT, alanine aminotransferase; AST, aspartate aminotransferase; ALP, alkaline phosphatase; TB, total bilirubin; γ-GT, γ-glutamyltranspeptidase; Cr, creatinine; LDH, dehydrogenase; CK, creatine kinase; CK-MB, creatine kinase-MB; TnT, troponin T; PT, prothrombin time; APTT, activated partial thromboplastin time; INR, international normalized ratio*.

Corticosteroid and immunoglobulin therapy was given to two of the three patients with pancreatitis. Less than half of the patients received corticosteroid and immunoglobulin therapy in the other two groups (Table 4). Almost all the patients received antibiotics because of the secondary infection in the ICU. It was noted that there were statistical differences in organ supports therapy (continuous renal replacement therapy *P* = 0.003 and mechanical ventilation *P* = 0.002) among the 55 patients with COVID-19.

**Table 4:** Treatments of patients with COVID-19.

**Variable**	**Patients of COVID-19 with pancreatitis**				**Patients of COVID-19 with elevatedpancreatic enzymes(*N* = 29)**	**Patients of COVID-19 without elevated enzymes (*N* = 23)**	***P***
	**P1**	**P2**	**P3**	**Overall**			
**Hospital treatment**, ***n*****(%)**							
Corticosteroid	N	Y	Y	2(66.7)	14(48.3)	11(47.8)	1
Immunoglobulin	Y	N	Y	2(66.7)	10(34.5)	5(21.7)	0.23
Antibiotics	Y	Y	Y	3(100)	29(100)	23(100)	-
Mechanical Ventilation	N	Y	Y	3(100)	21(72.4)	7(30.4)	0.002
CRRT	N	Y	Y	2(66.7)	13(44.8)	2(8.7)	0.003

*CRRT, continuous renal replacement therapy*.

### Three Critical COVID-19 Patients With Pancreatitis

**Figure 2:**
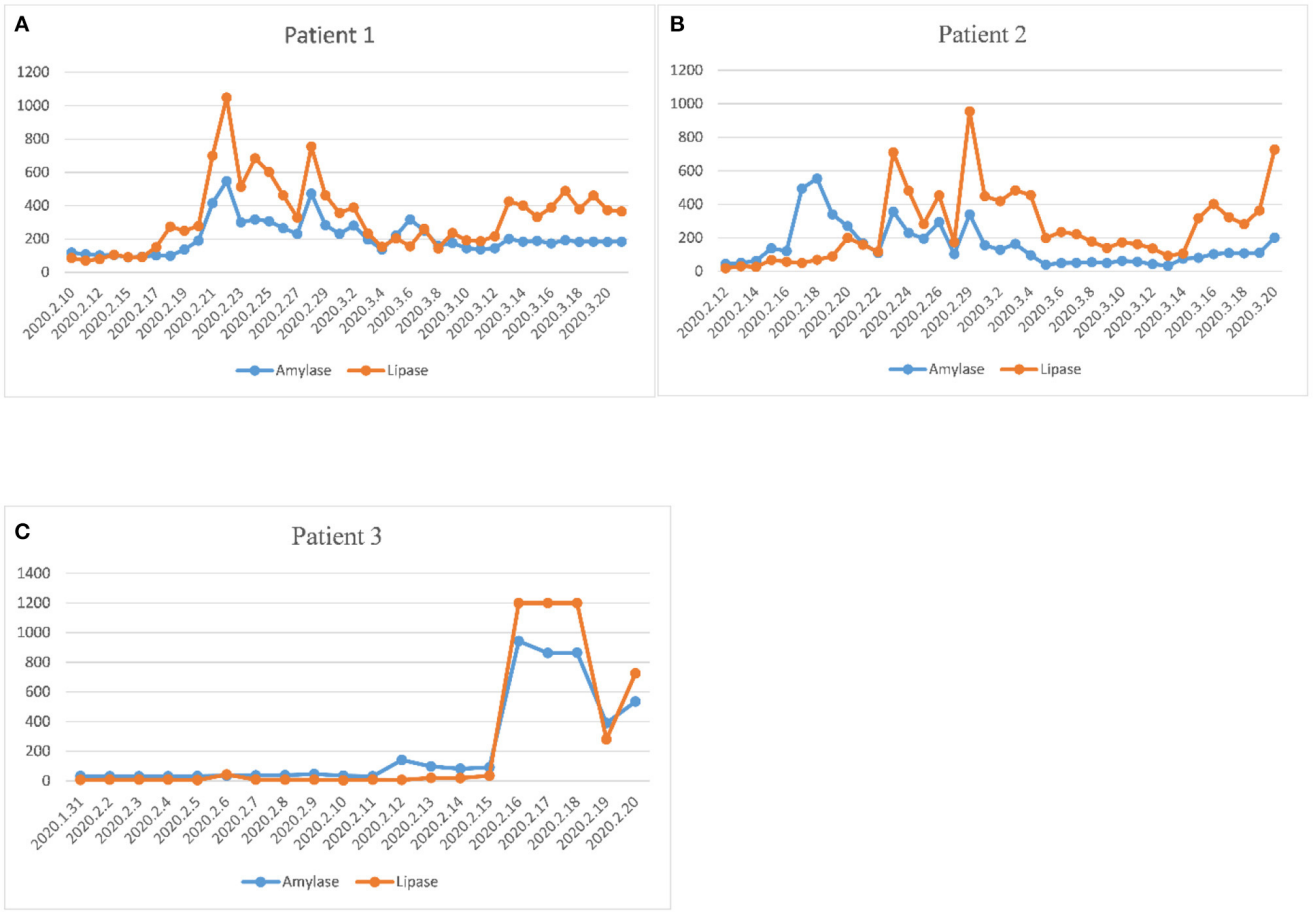
Evolution of plasma amylase and lipase during hospitalization in three pancreatitis patients. **(A)** The dynamic change of pancreatic enzymes in the first patient. **(B)** The dynamic change of pancreatic enzymes in the second patient. **(C)** The dynamic change of pancreatic enzymes in the third patient. The three patients with acute pancreatitis showed similar upward trends of amylase and lipase. The time to the peak of pancreatic enzymes was 11 days in the first patient, 17 days in the second patient, and 17 days in the third patient.

Among the 55 critically ill COVID-19 patients, three patients were diagnosed with acute pancreatitis. The trends of amylase and lipase in the three patients were plotted during hospitalization until discharge or death (Figure 2). The peak of amylase was 547 U/L in the first patient, 554 U/L in the second patient, 943 U/L in the third patient. The peak of lipase was 1,049 U/L in the first patient, 955 U/L in the second patient, >1,200 U/L in the third patient. The three patients with acute pancreatitis showed similar upward trends of amylase and lipase. The time to the peak of pancreatic enzymes was 11 days in the first patient, 17 days in the second patient, and 17 days in the third patient. Two of three patients died of severe multiple organ failure during hospitalization.

### Pancreatic Enzymes Elevation Was Associated With Several Influence Factors in Our Study

The results found that 136 out of the 277 cases had pancreatic enzymes elevation (Figure 1). The incidence of mild elevation (1–3 ULN) was 39.1%, and >3 × ULN was 10.9%. There were 32 patients with elevated pancreatic enzymes among the total recruited patients (*n* = 55). Twenty-nine patients with elevated pancreatic enzymes did not develop pancreatitis. This was confirmed by repeated transabdominal ultrasonography during hospitalization. Elevated pancreatic enzymes were seen in 58.2% of critically ill COVID-19 patients, and >3 × ULN in 40%. The median time to the amylase and lipase peaks (>3 × ULN) was 12 and 13 days, respectively. The peak value of amylase and lipase (>3 × ULN) was 819.2 ± 334.5 U/L and 355.8 ± 169.5 U/L, respectively (Table 5).

**Table 5:** Elevated amylase or lipase of 29 patients with COVID-19.

**Enzymes**		**Amylase**			**Lipase**	
	**Normal(10)**	**1–3 ULN(8)**	**>3 ULN(11)**	**Normal(1)**	**1–3 ULN(12)**	**>3 ULN(16)**
Cmax (Mean ± SD)	92.3 ± 23.0	220.6 ± 39.3	819.2 ± 334.5	46	121.2 ± 30.0	355.8 ± 169.5
Median (U/L)	94.5	222.5	690	46	123	295
Time to Max (d)	6.2 ± 5.0	14.9 ± 12.3	15.0 ± 11.0	20	9.9 ± 12.0	17.5 ± 10.6
Median (d)	6.5	16.5	12	20	7.5	13

*ULN, upper limit of normal; SD, standard deviation; Cmax, maximum concentration; Max, maximum. Twenty-nine patients with elevated pancreatic enzymes did not develop pancreatitis. Elevated pancreatic enzymes were seen in 58.2% of critically ill COVID-19 patients, and >3 × ULN in 40%*.

The outcomes of patients with or without elevated pancreatic enzymes were shown as Table 6. Patients with elevated pancreatic enzymes had higher rates of mortality (79.3 vs. 52.2%; *P* = 0.038), and lower rates of discharge (20.7 vs. 47.8%; *P* = 0.038) than the patients without elevated pancreatic enzymes.

**Table 6:** Association of elevated pancreatic enzymes with outcomes.

**Outcomes**	**Patients with elevated (*n* = 29)**	**Patients without elevated (*n* = 23)**	***P***
Death (*n*, %)	23(79.3)	12(52.2)	0.038
Discharge (*n*, %)	6(20.7)	11(47.8)	0.038

Although abnormally high pancreatic enzymes (>3 × ULN) are sensitive for the diagnosis of pancreatitis, there were several exceptions in our study. It is essential for clinicians to find the risk factors for increased pancreatic enzymes. This study analyzed the relevant possible influencing factors of patients during hospitalization. Patients with elevated pancreatic enzymes had a higher incidence of mechanical ventilation (*P* = 0.004) and kidney injury (*P* = 0.042) than patients without elevated pancreatic enzymes (Table 7). Multivariable logistic analysis show that pancreatic enzymes elevation was associated with mechanical ventilation (odds ratio = 10.202, *P* = 0.002) and acute kidney injury (odds ratio = 7.673, *P* = 0.014) (Table 8).

**Table 7:** Univariate analysis of elevated pancreatic enzymes in COVID-19 patients (*n* = 52).

**Influence factors**	**Group**	**Elevated (*n* = 29)**	**Without elevated (*n* = 23)**	***P***
Age	≥60	19	15	0.174
	<60	10	8	
Sex	1	18	17	0.368
	0	11	6	
Smoking history	1	2	7	0.079
	0	27	16	
Drinking history	1	1	4	0.125
	0	28	19	
Hypertension	1	13	9	0.68
	0	16	14	
Diabetes	1	6	4	0.785
	0	23	19	
COPD	1	0	0	-
	0	29	23	
Chronic nephrosis	1	1	2	0.436
	0	28	21	
Carcinoma	1	1	3	0.228
	0	28	20	
Hyperlipidemia	1	0	0	-
	0	29	23	
HCV	1	0	0	-
	0	29	23	
HBV	1	0	0	-
	0	29	23	
Fatty liver	1	7	7	0.612
	0	22	16	
Gallstone	1	4	6	0.271
	0	25	17	
Cholestasis	1	6	1	0.119
	0	23	22	
Mechanical ventilation	1	21	7	0.004
	0	8	16	
Shock	1	5	1	0.180
	0	24	22	
Kidney injury	1	13	4	0.042
	0	16	19	

*HBV, hepatitis B virus; HCV, hepatitis C virus*.

**Table 8:** Multivariate analysis of elevated pancreatic enzymes in COVID-19 patients (*n* = 52).

**Variable**	***B***	**OR**	**95%CI**	***P***
Mechanical ventilation	2.323	10.202	2.358–44.133	0.002
Kidney injury	2.038	7.673	1.521–38.714	0.014

*B, regression coefficient; OR, odds ratio; CI, confidence interval*.

## Discussion

SRAS-Cov-2 uses ACE2 receptors to invade the human body tissue cells (2). The pancreas can be a target of SARS-CoV-2 virus because it also expresses the ACE2 receptors (11). Several reports have shown that pancreatitis is one of the serious possible complications of COVID-19 disease (12–14). Furthermore, pancreatic enzymes elevation in COVID-19 patients has been reported in recent studies. Julia et al. reported that 2 of 71 patients (2.8%) had lipase elevation of >3 × ULN but none of the patients had acute pancreatitis (22). According to a study by Usman (23), 16.8% of patients have elevated levels of lipase enzyme (>3 × ULN). However, the two studies did not assess pancreas injury and relevant risk factors of elevated enzymes in COVID-19 patients were also not addressed. The present study was aimed to show the baseline characteristics and investigate the association of enzymes elevation with the outcomes and relevant risk factors in the first Chinese patients reported with critical COVID-19 disease.

In our study, elevated pancreatic enzymes were seen in 58.2% of critical-ill COVID-19 patients whereas >3 × ULN was reported in 40% of the patients. These results suggested that pancreatic enzyme elevation was common in critical COVID-19 patients. On the other hand, acute pancreatitis (AP) was rare. The development of pancreatitis is multifactorial consisting of susceptibility factors and associated injuries. The common causes of acute pancreatitis are alcohol, biliary obstruction, gall stones, and hypertriglyceridemia. It was found that one of the three patients with pancreatitis had gall stone and hypertriglyceridemia in our study. In the light of our clinical and review evidence, pancreatitis in the other two patients might be associated with the SARS-CoV-2 virus. Unfortunately, we did not carry out a postmortem to confirm if the SARS-CoV-2 virus actually existed in pancreas tissue. Therefore, further studies are needed to investigate the causes of AP in COVID-19.

A previous cohort study reported that COVID-19 patients with elevated pancreatic enzymes (>3 × ULN) have higher rates of ICU admission and intubation as compared with lower lipase levels (23). However, the study also lacked abdominal imaging to evaluate the pancreatic injury as a source of elevated enzymes. A higher incidence of intubation was also found in our patients. There are some factors that affect the prognosis of the COVID-19 patients, for example, male gender, older age, chronic kidney disease (24), hypercoagulability, and thrombotic complications (25). What's more, our study found that the elevation of pancreatic enzymes in critically ill COVID-19 patients have higher rate of mortality and lower incidence of discharge. This indicates that pancreatic enzymes elevation is also associated with adverse outcomes.

Serum pancreatic enzymes elevations can occur in many conditions not accused by pancreatitis, such as obstruction in gastroenteritis (26, 27), post-cholangiopancreatography (28), diabetes (29), several related drugs (dipeptidyl peptidase-4 inhibitors, alcohol) (30, 31), infection (HCV, HIV) (32, 33), multi-trauma (especially with head injury, blunt abdominal or pelvic trauma, liver injury) (34), biliary or gastrointestinal tumor, hepatocellular cancer, bowel cancer with liver metastases, renal injury (35, 36), and some critical-ill patients with mechanical ventilation or shock in ICU (35, 36).

To understand the relevant possible risk factors, we performed univariate analysis and multivariate analysis on the critical cases of COVID-19 with elevated pancreas enzymes and excluded pancreatic injury (Tables 7, 8). There were associations among pancreatic enzymes elevation, mechanical ventilation (*P* = 0.004) and kidney injury (*P* = 0.042). Subsequently, a multivariable logistic regression model was fit for pancreatic enzymes elevation among these variables showed signicant differences. Multivariable analysis confirmed that pancreatic enzymes elevation was associated with mechanical ventilation (odds ratio = 10.202, *P* = 0.002) and kidney injury (odds ratio = 7.673, *P* = 0.014). Therefore, in critically ill COVID-19 patients, oxygenation degration and kidney injury may be associated with abnormal pancreatic enzymes levels.

Glomerular filtration is primarily responsible for the clearance of serum amylase and lipase (37). However, some research studies pointed out that there was no much correlation between the raised amylase with acute kidney injury (38–40). Otherwise, Chen et al. found that the incidence of amylase and lipase elevation more than the normal upper limits were 35.7 and 26.2% in chronic renal failure, respectively (41). The finding of this study also shows that renal failure may be one of the risk factors in the occurrence of pancreatic enzymes elevation.

Inflammation caused by immune-medicated β-cell may have destroyed and caused the spill out of pancreatic enzymes through the exocrine pancreas in insulin-dependent diabetic conditions (42). This retrospective study showed that there was no association between preexisting diabetes and pancreatic enzymes elevation (*P* = 0.785). The same results were seen in other influencing factors including gallstone, fatty liver, cholestasis, related carcinoma, hypertriglyceridemia, HCV, and HBV infection (Table 7). Some scientific studies have pointed that elevations of pancreatic enzymes in ICU were related to septic shock and respiratory failure. Further, pancreatic hypo-perfusion can also be responsible for enzymes elevation. Critical illness may cause the pancreatic enzymes in the gut to enter into the submucosa and subsequently to the circulation as gut ischemia (35). Data from our study also demonstrated that oxygenation deterioration was associated with the elevated pancreatic enzymes in COVID-19 patients.

The limitation of our study included a relatively small sample size. The subjects were the first to be reported with critical COVID-19 disease in China at the beginning of 2020. However, the disease has been properly controlled at the later stage of the pandemic. Therefore, the results of this study were the initial state of critical COVID-19 disease at that time. Due to the shortage of medical resources, abdominal imaging could not be performed on every patient with elevated pancreatic enzymes. Additionally, due to the shortage of medical information, the examination was at the discretion of the ICU clinicians based on the patients' specific illness. In addition, the mortality could have been overestimated because of the lack of proper medical resources and information on COVID-19 during the early stage of the epidemic. It is recommended that further large-scale studies should be carried out to investigate the meaning of elevated pancreatic enzymes in critically ill patients.

In conclusion, it was found that, although the incidence of pancreatic enzymes elevation was more in critically ill COVID-19 patients, only a few progressed to acute pancreatitis (AP). It was also noted that critically ill COVID-19 patients with increased pancreatic enzymes could have developed poor clinical outcomes. Further, renal injury and oxygenation degradation could be associated with the elevation of the pancreatic enzymes. Therefore, this study analyzed relevant clinical data and articles retrospectively to provide the clinicians with a more comprehensive understanding for better clinical decisions for COVID-19 patients with elevated pancreatic enzymes.

## Data Availability Statement

The original contributions presented in the study are included in the article/supplementary material, further inquiries can be directed to the corresponding author/s.

## Ethics Statement

The studies involving human participants were reviewed and approved by the research ethics board of Wuhan Jinyintan Hospital. Written informed consent for participation was not required for this study in accordance with the national legislation and the institutional requirements.

## Author Contributions

PD wrote the main manuscript text. PD and BS collected the data. XL, XF, and HC analyzed the data. XZ and DZ revised the manuscript and gave final approval for the version to be published. All authors had contributed to the research conception and designed for the study. All authors have read and approved the manuscript.

## Conflict of Interest

The authors declare that the research was conducted in the absence of any commercial or financial relationships that could be construed as a potential conflict of interest.

## Publisher's Note

All claims expressed in this article are solely those of the authors and do not necessarily represent those of their affiliated organizations, or those of the publisher, the editors and the reviewers. Any product that may be evaluated in this article, or claim that may be made by its manufacturer, is not guaranteed or endorsed by the publisher.

## Abbreviations

COVID-19: coronavirus disease 2019
SARS-CoV-2: severe acute respiratory syndrome-related coronavirus 2
SARS-COV: severe acute respiratory syndrome-related coronavirus
MERS-COV: Middle East respiratory syndrome-related coronavirus
ACE-2: angiotensin-converting enzyme-2
ICU: intensive care unit
ULN: upper limit of normal
RT-PCR: real-time reverse transcription-polymerase chain reaction
KDIGO: Kidney Disease: Improving Global Outcomes
CT: computed tomography
MRI: magnetic resonance imaging
NLR: rate of neutrophils and lymphatic
PLR: rate of platelets and lymphatic
CRP: C-reactive protein
PCT: procalcitonin
ESR: erythrocyte sedimentation rate
IL-6: interleukin 6
ALB: albumin
ALT: alanine aminotransferase
AST: aspartate aminotransferase
ALP: alkaline phosphatase
TB: total bilirubin
γ-GT: γ-glutamyltranspeptidase
Cr: creatinine
LDH: dehydrogenase
CK: creatine kinase
CK-MB: creatine kinase-MB
TnT: troponin T
PT: prothrombin time
APTT: activated partial thromboplastin time
INR: international normalized ratio
CRRT: continuous renal replacement therapy
SD: standard deviation
Cmax: maximum concentration
Max: maximum
CMV: cytomegalovirus
HBV: hepatitis B virus
HCV: hepatitis C virus
HIV: human immunodeficiency virus
B: regression coefficient
OR: odds ratio
CI: confidence interval.

## References

[1] Hui DS Azhar E Madani TA Ntoumi F Kock R Dar O. The continuing 2019-nCoV epidemic threat of novel coronaviruses to global health - the latest 2019 novel coronavirus outbreak in Wuhan, China. Int J Infect Dis. (2020) 91:264–6. 10.1016/j.ijid.2020.01.009 31953166 PMC7128332

[2] Zhou P Yang X Wang X Hu B Zhang L Zhang W. A pneumonia outbreak associated with a new coronavirus of probable bat origin. Nature. (2020) 579:270–3. 10.1038/s41586-020-2012-7 32015507 PMC7095418

[3] Samavati L Uhal BD. ACE2, Much more than just a receptor for SARS-COV-2. Front Cell Infect Microbiol. (2020) 10:317. 10.3389/fcimb.2020.00317 32582574 PMC7294848

[4] Zhang H Penninger JM Li Y Zhong N Slutsky AS. Angiotensin-converting enzyme 2 (ACE2) as a SARS-CoV-2 receptor: molecular mechanisms and potential therapeutic target. Intensive Care Med. (2020) 46:586–90. 10.1007/s00134-020-05985-9 32125455 PMC7079879

[5] Xu X Chen P Wang J Feng J Zhou H Li X. Evolution of the novel coronavirus from the ongoing Wuhan outbreak and modeling of its Spike protein for risk of human transmission. Sci China Life Sci. (2020) 63:457–60. 10.1007/s11427-020-1637-5 32009228 PMC7089049

[6] Li F Li W Farzan M Harrison SC. Structure of SARS coronavirus spike receptor-binding domain complexed with receptor. Science. (2005) 309:1864–8. 10.1126/science.1116480 16166518

[7] Wan Y Shang J Graham R Baric RS Li F. Receptor recognition by novel coronavirus from Wuhan: an analysis based on decade-long structural studies of SARS. J Virol. (2020) 94:e127–20. 10.1128/JVI.00127-20 31996437 PMC7081895

[8] Zhao Y Zhao Z Wang Y Zhou Y Ma Y Zuo W. Single-Cell RNA expression profiling of ACE2, the receptor of SARS-CoV-2. Am J Respir Crit Care Med. (2020) 202:756–9. 10.1164/rccm.202001-0179LE 32663409 PMC7462411

[9] Hamming I Timens W Bulthuis MLC Lely AT Navis GJ Goor H. Tissue distribution of ACE2 protein, the functional receptor for SARS coronavirus. A first step in understanding SARS pathogenesis. J Pathol. (2004) 203:631–7. 10.1002/path.1570 15141377 PMC7167720

[10] Ding Y He L Zhang Q Huang Z Che X Hou J. Organ distribution of severe acute respiratory syndrome (SARS) associated coronavirus (SARS-CoV) in SARS patients: implications for pathogenesis and virus transmission pathways. J Pathol. (2004) 203:622–30. 10.1002/path.1560 15141376 PMC7167761

[11] Kusmartseva I Wu W Syed F Heide VVD Jorgensen M Joseph P. Expression of SARS-CoV-2 entry factors in the pancreas of normal organ donors and individuals with COVID-19. Cell Metab. (2020) 32:1041–51. 10.1016/j.cmet.2020.11.005 33207244 PMC7664515

[12] Amer H Mikkel W Klaus TK Pedersen UG Karstensen JG Novovic S. Coronavirus Disease-19 (COVID-19) associated with severe acute pancreatitis: case report on three family members. Pancreatology. (2020) 20:665–7. 10.1016/j.pan.2020.04.021 32387082 PMC7199002

[13] Alves AM Yvamoto EY Marzinotto MAN Teixeira AC Carriho FJ. SARS-CoV-2 leading to acute pancreatitis: an unusual presentation. Braz J Infect Dis. (2020) 24:561–4. 10.1016/j.bjid.2020.08.011 32961108 PMC7492046

[14] Meyers MH Main MJ Orr JK Obstein KL. A case of COVID-19-Induced acute pancreatitis. Pancreas. (2020) 49:e109–9. 10.1097/MPA.0000000000001696 33122538

[15] Wang F Wang H Fan J Zhang Y Wang H Zhao Q. Pancreatic injury patterns in patients with COVID-19 pneumonia. Gastroenterology. (2020) 159:367–70. 10.1053/j.gastro.2020.03.055 32247022 PMC7118654

[16] Huang C Wang Y Li X Ren L Zhao J Hu Y. Clinical features of patients infected with 2019 novel coronavirus in Wuhan, China. Lancet. (2020) 395:497–506. 10.1016/S0140-6736(20)30183-5 31986264 PMC7159299

[17] National Health Commission of the People's Republic of China. The seventh edition of the COVID-19 diagnosis and treatment plan. (2020). Available online at: http://www.nhc.gov.cn/yzygj/s7652m/202003/a31191442e29474b98bfed5579d5af95.shtml (accessed March 4, 2020).

[18] Banks PA Bollen TL Dervenis C Gooszen HG Johnson CD Sarr MG. Classification of acute pancreatitis-2012: revision of the Atlanta classification and definitions by international consensus. Gut. (2013) 62:102–11. 10.1136/gutjnl-2012-302779 23100216

[19] KDIGO. 2012 Kidney Disease: Improving Global Outcomes (KDIGO) clinical practice guideline for acute kidney injury (AKI). (2012). Available online at: https://kdigo.org/guidelines/acute-kidney-injury/ (accessed March 4, 2014).

[20] Cecconi M De Backer D Antonelli M Beale R Bakker J Hofer C. Consensus on circulatory shock and hemodynamics moitoring. Task force of the European Society of Intensive Care Medicine. Intensive Care Med. (2014) 40:1795–815. 10.1007/s00134-014-3525-z 25392034 PMC4239778

[21] Standl T Annecke T Cascorbi I Heller AR Sabashnikov A Teske W. The nomenclature, definition and distinction of types of shock. Dtsch Arztebl Int. (2018) 115:757–68. 10.3238/arztebl.2018.0757 30573009 PMC6323133

[22] Julia MB Jin DX Grover AS Redd WD Zhou JC Hathorn KE. Lipase elevation in patients with COVID-19. Am J Gastrornterol. (2020) 115:1286–8. 10.14309/ajg.0000000000000732 32496339 PMC7288768

[23] Barlass U Wiliams B Dhana K Adnan D Khan SR Mahdavinia M. Marked elevation of lipase in COVID-19 disease: a cohort study. Clin Transl Gastroenterol. (2020) 11:e00215. 10.14309/ctg.0000000000000215 32764201 PMC7386395

[24] Fang XY Li S Yu H Wang PH Zhang Y Chen Z. Epidemiological, comorbidity factors with severity and prognosis of COVID-19: a systematic review and meta-analysis. Aging. (2020) 12:12493–12503. 10.18632/aging.103579 32658868 PMC7377860

[25] Jiang MS Mu JX Shen SL Zhang H. COVID-19 with preexisting hypercoagulability digestive disease. Front Med. (2020) 7:587350. 10.3389/fmed.2020.587350 33521013 PMC7838325

[26] Lott JA Patel ST Sawhney AK Kazmierczak SC Jr JEL. Assays of serum lipase: analytical and clinical considerations. Clin Chem. (1986) 32:1290–302. 10.1093/clinchem/32.7.1290 2424637

[27] Diani G Poma G Novazzi F Zanirato S Porta C Moroni M. Increased serum lipase with associated normoamylasemia in cancer patients. Clin Chem. (1998) 44:1043–5. 10.1093/clinchem/44.5.1043 9590380

[28] Gottlieb K Sherman S Pezzi J Esber E Lehman GA. Early recognition of post-ERCP pancreatitis by clinical assessment and serum pancreatic enzymes. Am J Gastroenterol. (1996) 91:1553–7. 8759660

[29] Malloy J Gurney K Shan K Yan P Chen S. Increased variability and abnormalities in pancreatic enzyme concentrations in otherwise asymptomatic subjects with type 2 diabetes. Diabetes Metab Syndr Obes Targets Ther. (2012) 5:419–24. 10.2147/DMSO.S34241 23269874 PMC3529626

[30] Lando HM Alattar M Dua AP. Elevated amylase and lipase levels in patients using glucagonlike peptide-1 receptor agonists or dipeptidylpeptidase-4 inhibitors in the outpatient setting. Endocr Pract. (2012) 18:472–7. 10.4158/EP11290.OR 22440997

[31] Asvesta S Pantopoulos K Arzoglou PL. Lipase activity and properties in serum of chronic alcoholics. Ann Biol Clin. (1988) 46:435–38. 3189969

[32] Yoffe B Bagri AS Tran T Dural AT Shtenberg KM Khaoustov VI. Hyperlipasemia associated with hepatitis C virus. Dig Dis Sci. (2003) 48:1648–53. 10.1023/A:1024744613671 12924663

[33] Argiris A Mathur-Wagh U Wilets I Mildvan D. Abnormalities of serum amylase and lipase in HIV-positive patients. Am J Gastroenterol. (1999) 94:1248–52. 10.1111/j.1572-0241.1999.01074.x 10235202

[34] Hameed AM Lam VWT Please HC. Significant elevations of serum lipase not caused by pancreatitis: a systematic review. HPB. (2015) 17:99–112. 10.1111/hpb.12277 24888393 PMC4299384

[35] Denz C Siegel L Lehmann KJ Dagorn JC Fiedler F. Is hyperlipasemia in critically ill patients of clinical importance? An observational CT study. Intensive Care Med. (2007) 33:1633–6. 10.1007/s00134-007-0668-1 17497124

[36] Muniraj T Dang S Pitchumoni CS. Pancreatitis or not?-Elevated lipase and amylase in ICU patients. J Crit Care. (2015) 30:1370–5. 10.1016/j.jcrc.2015.08.020 26411523

[37] Junge W Malyusz M Ehrens HJ. The role of the kidney in the elimination of pancreatic lipase and amylase from blood. J Clin Chem Clin Biochem. (1985) 23:387–92. 10.1515/cclm.1985.23.7.387 2413160

[38] Frank B Gottlieb K. Amylase normal, lipase elevated: is it pancreatitis? A case series and review of the literature. Am J Gastroenterol. (1999) 94:463–9. 10022647 10.1111/j.1572-0241.1999.878_g.x

[39] Zachee P Lins RL De Broe ME. Serum amylase and lipase values in acute renal failure. Clin Chem. (1985) 31:1237. 10.1093/clinchem/31.7.1237 2408790

[40] Smith RC Southwell KJ Chesher D. Should serum pancreatic lipase replace serum amylase as a biomarker of acute pancreatitis? ANZ J Surg. (2005) 75:399–404. 10.1111/j.1445-2197.2005.03391.x 15943725

[41] Chen CC Wang SS Chen TW Jap TS Chen SJ Jeng FS. Serum procarboxypeptidase B, amylase and lipase in chronic renal failure. J Gastroenterol Hepatol. (1996) 11:496–9. 10.1111/j.1440-1746.1996.tb00297.x 8743924

[42] Semakula C Vandewalle CL Schravendijk CFV Sodoyez JC Schuit FC Foriers A. Abnormal circulating pancreatic enzyme activities in more than twenty-five percent of recent-onset insulindependent diabetic patients: association of hyperlipasemia with high-titer islet cell antibodies. Belgian Diabetes Registry. Pancreas. (1996) 12:321–33. 10.1097/00006676-199605000-00001 8740397

